# Thermal conductivity and viscosity measurements of ethylene glycol-based Al_2_O_3 _nanofluids

**DOI:** 10.1186/1556-276X-6-221

**Published:** 2011-03-15

**Authors:** María José Pastoriza-Gallego, Luis Lugo, José Luis Legido, Manuel M Piñeiro

**Affiliations:** 1 Departamento de Física Aplicada, Facultade de Ciencias, Universidade de Vigo, Campus Universitario s/n, E-36310, Vigo, Spain

## Abstract

The dispersion and stability of nanofluids obtained by dispersing Al_2_O_3 _nanoparticles in ethylene glycol have been analyzed at several concentrations up to 25% in mass fraction. The thermal conductivity and viscosity were experimentally determined at temperatures ranging from 283.15 K to 323.15 K using an apparatus based on the hot-wire method and a rotational viscometer, respectively. It has been found that both thermal conductivity and viscosity increase with the concentration of nanoparticles, whereas when the temperature increases the viscosity diminishes and the thermal conductivity rises. Measured enhancements on thermal conductivity (up to 19%) compare well with literature values when available. New viscosity experimental data yield values more than twice larger than the base fluid. The influence of particle size on viscosity has been also studied, finding large differences that must be taken into account for any practical application. These experimental results were compared with some theoretical models, as those of Maxwell-Hamilton and Crosser for thermal conductivity and Krieger and Dougherty for viscosity.

## Introduction

Improving the efficiency of energy production and consumption has undoubtedly become one of the most important global problems that will have to be faced during the next decades. Some of the particular concerns related with this main problem include the quantification and control of global climate change due to the emissions of greenhouse gases, or the expected decline in global oil production [1]. Considering the rapid increase in energy demand worldwide, intensifying heat transfer processes and reducing energy losses due to ineffective use have become increasingly important tasks. Nanoscience and nanotechnology are expected to play a significant role in revitalizing the traditional energy industries and stimulating the emerging renewable energy industries [2,3]. Nanofluids, in which nano-sized particles are suspended in liquids, have emerged as a potential candidate for the tailoring and production of heat transfer fluids. It is known that these new fluids enhance thermal conductivity of the base liquid, although the underlying nature of this effect still remains controversial. Moreover, nanofluids were found to be very stable due to the small size of the particles and the small volume fraction of the particles needed for heat transfer enhancement [4].

When the nanoparticles are properly dispersed, nanofluids can offer numerous benefits [5-7] besides the anomalously high effective thermal conductivity, such as improved heat transfer and stability, microchannel cooling without clogging, the possibility of miniaturizing systems scalings, or reduction in pumping power, among others. Thus, nanofluids have a wide range of industrial, engineering, and medical applications in fields ranging from transportation, micromechanics, heating, ventilating and air-conditioning systems, biomolecules trapping, or enhanced drug delivery [3,8].

When studying this type of systems, one of the variables that must be considered carefully in first place is the sample polydispersity because usually, the average particle size values declared to characterize samples are only rough approximations, and definitely, a non-negligible size distribution is always present for real samples, producing noticeable changes in thermal behavior. Once the dry nanoparticles are well characterized, the stability of the suspensions must then be ensured. The measurement of zeta potential and the use of UV/Vis spectrophotometry represent reliable probes to quantify stability [9-11]. Usually, the dispersion in the base fluid is obtained using techniques such as mechanical stirring, ultrasound probes, or the combination of both, but also in this case, there are no clear guidelines about the most reliable method to achieve stability and avoid sedimentation. The recommended sonication times vary for the same nanofluid according to different authors, and the effect on the size and distribution of aggregates is seldom discussed [12,13]. Visual technique controls may be discarded in this context for their lack of reproducibility.

Although the determination of thermal conductivity has focused most efforts, it is believed that viscosity is as critical as thermal conductivity in engineering systems that entail fluid flow [8,14-16]. Pumping power is proportional to the pressure drop, which in turn is related to fluid viscosity. In laminar flow, the pressure drop is directly proportional to the viscosity. Both viscosity and thermal conductivity of nanofluids are known to undergo anomalous enhancements, but more thorough investigations should be carried out on these properties because a good deal of controversy and remarkable inconsistencies have been reported in this emerging subject [17]. The monograph published by Das et al. [4] represents a reference study about nanofluids, including a wide literature survey, which is indicative of the efforts done in the last few years. A recent collective study [18] intended to establish a benchmark for thermal conductivity measurements by comparing the results obtained from a common sample delivered to many reference laboratories. The results yielded differences between 5% and 10% for data of water and PAO-based samples from different sources. In other recent studies concerning thermophysical characterization of nanofluids, Das et al. [19] and Eastman et al. [15] presented a good account about nanotubes and the role of the contact resistance in the thermal transport of nanofluids, besides addressing the issues about thermal conductivity and viscosity of oxide nanoparticle-based and metallic nanofluids. Wang and Mujumdar [11] presented an overview focused on heat transfer characteristics using nanofluids, and Murshed et al. [8] remarked that it is imperative to conduct detailed research in order to confirm the effects of particle size, shapes, clustering of particles, and temperature on the effective thermal conductivity of a wide range of nanofluids and added that it is necessary to develop more comprehensive models, based on first principles, with the aim of accounting for the enhanced thermal conductivity of nanofluids. Li et al. [20] also discussed the preparation and characterization of nanofluids, a subject that unfortunately has not received the necessary attention so far but plays a key role. Wen et al. [2] and Murshed et al. [21] insisted on the need of studies about other properties such as viscosity, wetting behavior, thermal diffusivity, convective heat transfer coefficients, and viscosity; finally, Özerinç et al. [22] summarized the research in nanofluid thermal conductivity from experimental and theoretical investigations.

In this general context, the objective of this article was to study nanofluids composed by alumina (Al_2_O_3_) nanoparticles dispersed in ethylene glycol in a concentration ranging up to 25% in weight fraction. Two different sets of samples were considered, one of them obtained from dispersion of different brands of commercial dry nanopowder and the second obtained from dispersion of a dry nanopowder obtained by centrifuged and dried of a commercial dispersion. The characteristics of the dry powder, stability, size distribution, and *Z *potential are discussed in each case. Then, the thermal conductivity and viscosity of the nanofluids have been determined experimentally between 283.15 K and up to 323.15 K.

From a theoretical point of view, it was Maxwell [23] who first proposed a theory to account for the enhancement produced in the thermal conductivity of a fluid by the presence of suspended colloidal particles. Unfortunately, the classical models on suspensions give an insufficient understanding of the formulation and thermophysical profile of nanofluids, thus limiting their potential applications. Although it is widely agreed now that the initial thermal conductivity enhancements reported were by far too optimistic, a reliable theory connecting the molecular structure and the macroscopic transport properties of nanofluids is not available yet, so a considerable effort for the determination of accurate and reproducible experimental data for this type of suspensions is essential. The results presented in this work have been compared with other reported experimental values and with various theoretical models proposed for the prediction of the thermal conductivity and viscosity of nanofluids. Concerning experimental and theoretical studies on alumina nanoparticles dispersed in ethylene glycol, the works studying the effect of temperature by Timofeeva et al. [24] and Beck et al. [25-27] must be cited. Alternatively, Beck et al. [28] have studied the effect of particle size on thermal conductivity and Timofeeva et al. [24,29] considered the effect of particle shape and pH on this property and also on viscosity, from both experimental and theoretical perspectives. Timofeeva et al. have drawn attention on the fact that evaluation of nanofluids for a particular application requires proper understanding of all their characteristics and thermophysical properties of nanoparticle suspensions.

## Experimental

### Sample preparation and characterization

Two sets of different samples of ethylene glycol-based Al_2_O_3 _nanofluids were used. The first of them, S1, was prepared by dispersing dry Al_2_O_3 _nanoparticles in ethylene glycol (Aldrich, St. Louis, MO, USA, 99%). The nanoparticles were supplied by Nanophase, with a declared diameter distribution *D *= 40-50 nm and a crystal phase composition of 70:30 γ and δ phases, respectively. Samples S2 were prepared using Al_2_O_3 _nanoparticles supplied by Aldrich dispersed in water (10% weight fraction), with a limiting value of *D *< 20 nm. This original dispersion was centrifuged and washed repeatedly with absolute ethanol, and the obtained solid was dried and redispersed in ethylene glycol. The powder sample was in every case dispersed into a predetermined volume of the base fluid to obtain the desired weight fraction. Values up to 20 wt.% for viscosity, and up to 25% for thermal conductivity measurements were prepared using a Mettler AE-240 electronic balance (Mettler-Toledo, Columbus, OH, USA), whose accuracy is 5 × 10^-5 ^g.

**Figure 1:**
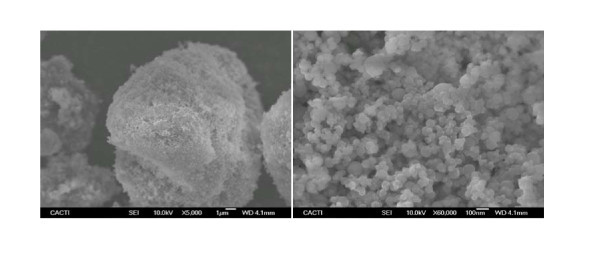
**SEM image of S1 dry Al_2_O_3_nanopowder at two magnifications**. **a **× 5,000; **b **× 60,000.

All products were used without any purification, and no dispersants or surfactants were used to stabilize the samples. As it has been shown that the size, shape, and composition of nanoparticles strongly influence their thermophysical profile, the first step to obtain a precise characterization of the samples was the analysis of the dry nanoparticles used in the preparation of S1 and S2. In this case, the scanning electron microscopy [SEM] technique was used, and the images were obtained with a JEOL JSM-6700F field emission gun-SEM, (JEOL, Tokyo, Japan), operating at an acceleration voltage of 20 kV in backscattering electron image (yttrium aluminium garnet-type detector). This device incorporates an energy-dispersive X-ray spectrometer that was used to chemically characterize the samples. SEM samples were prepared by deposition of the nanopowder on top of a carbon substrate, coated with a thin (approximately 20 nm) carbon layer. The pictures in Figure 1 show that under atmospheric condition, the nanopowder forms close agglomerates of micrometers in size (Figure 1a). A magnification of these aggregates (Figure 1b) allows identifying the individual nanoscale size particles on the agglomerate surface. The shape of the individual nanoparticles is nearly spherical.

As described in a previous work [10], the use of an ultrasonic homogenizer improves nanofluid stability over other alternatives available to disperse the nanoparticles, and so a (U.S.int) BandelinSonoplus HD 2200 was used (Bandelin Electronic, Berlin, Germany), with typical sonication times of 16 min. In order to check the morphology and size distribution of the fluid samples, transmission electron microscope technique was used [10]. An estimate of the size distribution in each case was obtained using ImageTool freeware software http://www.digitalimagetool.com. The volume-weighted average diameter values computed were *D *= 43 ± 23 nm for S1 and *D *= 8 ± 3 nm for S2 [10]. More details about sample preparation and characterizations are given in [10,30].

### Thermal conductivity and viscosity measurements of nanofluids

Once both samples have been adequately characterized, the following step is to determine the thermal conductivity and viscosity of the nanofluids. The transient hot-wire method was first suggested in 1931 to measure the absolute thermal conductivity, and ever since many authors have contributed to improve the method, making it more accurate. With the development of modern electronic instrumentation and use of a proper theoretical basis, this method has evolved to be one of the most accurate techniques of determining the thermal conductivity of fluids, including nanofluids [8,31]. The advantage of this method is connected with its success to nearly completely avoid natural convection effects. In addition, this method is fast and its conceptual design is simple when compared to other techniques. Thermal conductivity data were measured in this case using the Decagon devices KD2 Pro Thermal Properties Analyzer (Decagon Devices Inc., Pullman, WA, USA). This apparatus meets the standards of ASTM D5334 and IEEE 442-1981 regulations. Its principle of measurement is based on the transient hot-wire source approach, and it has been used successfully for nanofluids by several authors [29,32-34]. It basically comprises a readout unit and a single-needle sensor that is inserted into the fluid sample. The thermal probe (1.27-mm diameter, 60-mm length), containing a heating element and a thermoresistor, should be inserted into the sample vertically, rather than horizontally, with the aim of minimizing the possibility of inducing convection. The measurement is made by heating the probe within the sample while simultaneously monitoring the temperature change of the probe. A single reading generally takes 2 min. The first 90 s are used to ensure temperature stability, after which the probe is heated for 30 s using a controlled current intensity. The thermistor measures the changing temperature while the microprocessor stores the data. At the end of the reading, the thermal conductivity of the fluid is computed using the temperature difference versus time data based on a parameter-corrected version of the temperature model given by Carslaw and Jaeger [35] for an infinite line heat source with constant heat output and zero mass in an infinite medium. Before and after analysis of the nanofluid samples, the accuracy of the probe was carefully checked on pure water, ethylene glycol, and a standard sample of glycerol of well-known thermal conductivity. Approximately 15 cm^3 ^of the sample to be analyzed was sealed in a glass sample vial. The probe was then inserted vertically into the sample via a purpose-made port in the lid of the vial. The sealed vial was then fully immersed in a temperature-controlled water bath, model Grant GD200, (Grant Instruments, Cambridge, UK), and allowed to thermostatize. Once the sample reached the required temperature, 15 more minutes were allowed to go before carrying out the measurement to ensure complete thermal equilibration. At least four measurements were taken at each temperature, with a delay of at least 15 min between each other, to ensure reproducibility. The uncertainty of the thermal conductivity was estimated from the standard deviations of experimental data and departures from literature values of the cited reference fluids, and was estimated to be lower than 3%.

Viscosity measurements of alumina nanofluids were performed using a Schott rotational viscometer (Cole Parmer, Vernon Hills, IL, USA), equipped with a spindle of coaxial cylindrical geometry (LCP) equipped with a stainless steel flow jacket. This viscometer is a controlled shear rate instrument. By using a multiple-speed transmission and interchangeable spindles, a variety of viscosity ranges can be measured, enhancing device versatility. Flow behavior of nanofluids was tested at a shear rate of 123 s^-1^. The LCP adaptor holds a sample volume of 16-18 ml and is connected to a PolyScience fluid circulation bath (PolyScience, Niles, IL, USA), that controls temperature measured inside the cell with a PT100 probe that ensures an uncertainty of 0.05 K. The estimated uncertainty in viscosity using this device is guaranteed to within ± 1%.

## Results and discussion

### Thermal conductivity

**Figure 2:**
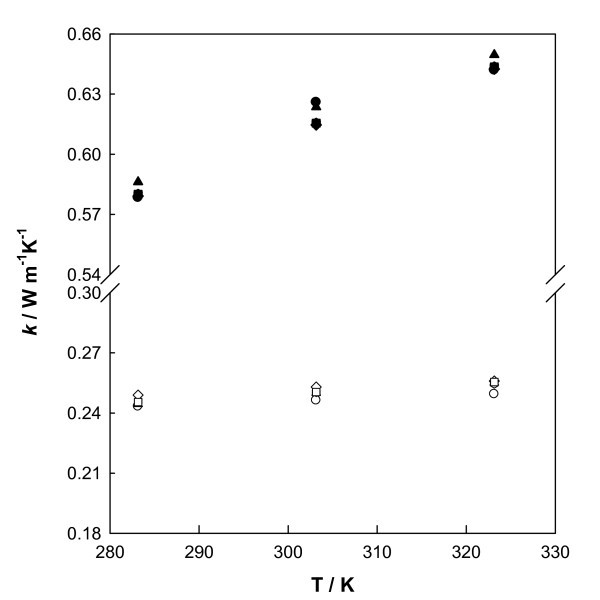
**Comparison of thermal conductivity values**. Values obtained in this work (*filled circle*, water; *empty circle*, EG) and several literature values for water (filled *triangle *[38]; *filled square *[37]; *filled diamond *[41]; *downturned triangle *[36]) and EG (*ex *[52]; *empty diamond *[39]; *empty square *[42], empty *triangle *[38]).

The experimental thermal conductivities at atmospheric pressure from 283.15 K to 323.15 K for pure ethylene glycol and water were determined first and are presented in Table 1. A comparison between our data and those from literature [36-42] is displayed graphically in Figure 2. Overall average deviation of 1.8% is obtained for ethylene glycol and 0.8% for water. An inspection of the data presented in Figure 2 shows that our results are in agreement with literature values within the estimated experimental uncertainty.

**Table 1:** Experimental thermal conductivity for ethylene glycol and water

EG		H_2_O	
***T *(K)**	***k *(W m^-1 ^K^-1^)**	***T *(K)**	***k *(W m^-1 ^K^-1^)**

283.15	0.2433	283.15	0.5784

303.15	0.2463	303.15	0.6259

323.15	0.2494	323.15	0.6345

**Figure 3:**
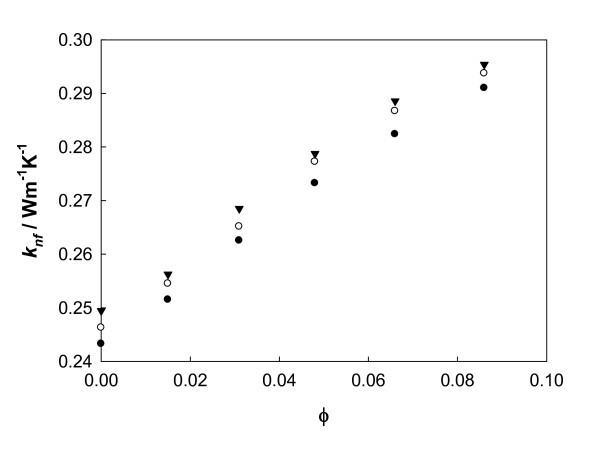
**Experimental measured thermal conductivity**. Alumina nanofluids in EG versus volume fraction concentration at different temperatures: 283.15 K (*filled circle*); 303.15 K (*empty circle*), and 323.15 K (*downturned triangle*).

The thermal conductivity enhancement of five different ethylene glycol-based Al_2_O_3 _nanofluids corresponding to the denoted S1 samples has been measured at 283.15 K, 303.15 K, and 323.15 K. The volume fraction, *ϕ*, varied between 1.5% and 8.6% estimated from the densities of the pure liquid, determined in our laboratory with an Anton Paar DMA 4500 vibrating tube densimeter (Anton Paar, Graz, Österreich), and the bulk solid oxide [41]. The experimental thermal conductivities of alumina nanofluids, *k*_nf_, at several temperatures are presented in Table 2 as a function of volume fraction. At the tested concentrations, thermal conductivity increases with nanoparticle volume fraction, *ϕ*, as shown in Figure 3. This behavior is in agreement with Timofeeva et al. [24,29] for different particle shapes, including spheres, and with those reported by Beck et al. [25] and Wang et al. [43] for nanofluids consisting of ethylene glycol with 20- and 28-nm alumina nanoparticles, respectively. Concerning temperature dependence, the thermal conductivity of all nanofluids also increases with temperature. As observed, the addition of nanopowder systematically increases the thermal conductivity of the nanofluid as compared with the pure fluid. If Table 2 is analyzed, we can conclude that this enhancement for a given nanofluid is nearly temperature-independent, as Peñas et al. [38] have also stated. Average enhancements values from 3% at the lowest volume fraction up to 19% for the highest concentration are found, showing good agreement (average 1% deviation) with the data from Timofeeva et al. [24] at 296.15 K for suspensions prepared from 40-nm alumina nanoparticles. However, the S1 sample data reported here and those from [24] with 11-, 20-, and 40-nm nominal sizes do not show the same trend as reported by Beck et al. [28] in their study on the effect of particle size on alumina nanofluids in ethylene glycol. This may be due to the different pH of the samples studied [29], an effect that has been cited to have an influence on this property.

**Table 2:** Experimental values of the thermal conductivity of nanofluids based on EG (S1 samples)

*ϕ*	*k*_nf _(W m^-1 ^K^-1^)		
	
	283.15 K	303.15 K	323.15 K
0.000	0.2433	0.2463	0.2494

0.015	0.2515	0.2545	0.2562

0.031	0.2626	0.2652	0.2685

0.048	0.2733	0.2773	0.2788

0.066	0.2824	0.2867	0.2886

0.086	0.2910	0.2938	0.2954

In the past decade, many efforts have been made to theoretically estimate the enhancement of thermal conductivity of nanofluids, and a rather large number of models have been proposed. However, and despite the efforts to account for different physical effects, none of these models can be used with enough generality. The classical Maxwell model [23] for thermal conductivity was proposed to predict the thermal conductivity of homogeneous liquid/solid suspensions with relatively large and spherical particles. This model has been applied here in its original formulation.(1)

where *k*_nf_, *k*_p_, and *k*_0 _stand for the thermal conductivity of the nanofluid, solid particles, and bulk liquid, respectively, and *ϕ *is the particle volume fraction (vol.%). For the thermal conductivity of the particles, we used tabulated values [41] for the bulk solid, *k*_Al2O3 _= 36 W m^-1 ^K^-1 ^(polycrystalline).

Many other models were proposed based on the traditional Maxwell formulation, considering the influence of factors as particle diameter, surface area, shape, Brownian motion, or solid/fluid interfacial effects. Wang and Mujumdar [11] extensively reviewed different nanofluid thermal conductivity theories, beginning with the adaptation by Hamilton and Crosser [44] of the classical Maxwell model.

The effects of solid/fluid interface are very important in suspensions. The nanolayer between the nanoparticles and the base fluid may be a dominant factor influencing the thermal conductivity of nanofluids. Current research on nanofluids indicates that the enhancement of thermal conductivity might be due to the ordered layering of liquid molecules near the solid particles, and some models taking this effect into account have been developed [20]. Nevertheless, it is beyond the goal of this work to compare our experimental data with an extensive review of models. Moreover, as was pointed out elsewhere [11] for dilute concentrations, there is little difference between the classical Maxwell model and other more sophisticated theories.

**Figure 4:**
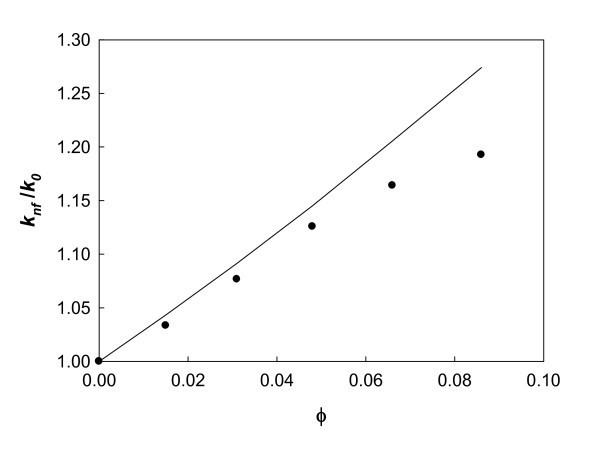
**Enhancement in the thermal conductivity at 303.15 K**. Alumina nanofluids as a function of the volume fraction of nanoparticles. *Solid line*, Prediction of Maxwell model of Equation 1.

The experimental values of the thermal conductivity together with the predictions based on Equation 1 for the different nanofluids studied are represented in Figure 4 at 303.15 K, and similar results have been obtained at all temperatures. As can be seen, the Maxwell model overpredicts the experimental enhancement of the thermal conductivity. This behavior is also found for several sets of data of Al_2_O_3_/water nanofluids examined in [11], with also larger deviations appearing at higher volume fractions. As a conclusion, and in coincidence with the common opinion, it is still necessary to develop further investigation about thermal transfer processes in nanofluids considering some of the variables cited above. Nevertheless, as a first step, it is even more necessary to concentrate efforts on a very accurate experimental determination, controlling all properties involving in nanofluids and standardizing the characterization and preparation of new nanofluids, keeping in mind the objective of obtaining a perfectly reliable reproducibility in sample preparation at a first stage and then the same reproducibility in thermophysical property determination as that currently achieved when dealing with classical fluids and solutions.

### Viscosity

Viscosity describes a fluid internal resistance to flow and, in the case of nanofluids, depends on the morphology and size of nanoparticles. Although some studies indicate non-Newtonian behavior of nanofluids, specially at low shear rate, Wang et al. [43] and Chen et al. [45] indicated for Al_2_O_3_/EG nanofluids a Newtonian behavior at relatively high shear rates, and the value stated in this work (*γ *= 123 s^-1^) lies in that range. Experimental viscosity values at atmospheric pressure and at 5 K intervals, from 283.15 K to 323.15 K, for S1 and S2 are listed in Table 3. Experimental results for pure ethylene glycol were compared with those reported by Sun and Teja [39] and by Chen et al. [45], finding a good agreement, with an average deviation of 1% and 2%, respectively.

**Table 3:** Experimental viscosity values, *η *(mPa·s), for nanofluids based on EG constituted by S1 and S2 samples

*ϕ*	*T *(K)							
	
	283.15	288.15	293.15	298.15	303.15	308.15	313.15	323.15
S1 samples								

0.000	35.44	28.00	21.89	17.25	13.86	11.64	9.62	7.21

0.005	37.30	29.54	23.61	18.35	14.48	12.16	10.17	7.51

0.010	40.29	31.54	25.22	19.91	15.87	13.55	11.21	8.26

0.015	43.21	33.75	26.61	21.05	16.75	14.27	11.89	8.73

0.021	46.67	36.20	28.51	22.69	18.18	15.16	12.53	9.27

0.031	51.90	39.79	31.99	25.64	20.55	17.00	13.79	10.44

0.048	65.43	49.41	38.07	30.46	24.31	20.32	16.80	12.40

0.066	81.51	61.27	47.70	37.86	30.87	25.35	21.50	15.41

S2 samples								

0.000	35.44	28.00	21.89	17.25	13.86	11.64	9.62	7.21

0.005	40.54	32.01	24.50	19.46	15.76	13.13	10.84	8.10

0.010	46.07	34.98	27.06	21.57	17.67	14.52	12.05	8.96

0.015	53.50	40.85	30.44	23.78	19.41	15.85	13.01	9.47

0.021	61.35	46.86	35.62	27.80	22.31	18.20	14.94	11.02

0.031	75.19	57.48	43.80	33.92	27.02	21.80	18.27	13.26

**Figure 5:**
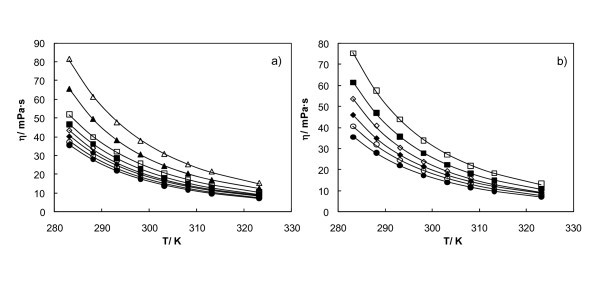
**Dynamic viscosities for both Al_2_O_3_/EG nanofluids versus temperature**. S1 samples (**a**) and S2 samples (**b**). Experimental points at different volume fractions: EG (*filled circle*), 0.005 (*empty circle*), 0.010 (*filled diamond*), 0.015 (*empty diamond*), 0.021 (*filled square*), 0.031 (*empty square*), 0.048 (*filled triangle*), 0.066 (*empty triangle*), Vogel-Fulcher-Tammann equation (*solid line*).

Concentrations from 1.7% to 20% in weight fraction, corresponding to volume fractions from 0.005 to 0.065, were considered for nanofluids using S1 samples, while concentrations from 1.7% to 10% in weight fraction, corresponding to volume fractions from 0.005 to 0.03, were measured for the S2 samples. The viscosity decreases significantly with temperature, as usual, as represented in Figure 5.

A large number of methods have also been developed to describe the dependence of viscosity for different fluids with temperature. Among them, the following modification of Andrade's equation, also known as three-coefficient Vogel-Fulcher-Tammann equation, was proposed:(2)

where *η *is the dynamic viscosity, *T *is the temperature, and *A*, *B*, and *T*_0 _are adjustable parameters. The ratio of parameters *B *and *T*_0 _is also known as Angell strength parameter [46]. The values obtained from *A*, *B*, and *C *are gathered in Tables 4 and 5 for different nanofluids. The average standard deviation of these correlations is 0.3 mPa s for both S1 and S2 samples, the maximum being 0.7 and 0.5 mPa s, respectively. The goodness of this fit can also be seen in Figure 5.

**Table 4:** Coefficients *A*, *B*, *T*_0_, and standard deviation, *s*, from Vogel-Fulcher-Tammann equation for S1 Al_2_O_3_/EG nanofluids at different volume concentration, ***ϕ***

	*ϕ*							
	
	0.000	0.005	0.010	0.015	0.021	0.031	0.048	0.066
*A*	-3.694	-3.632	-2.381	-1.702	-3.450	-3.302	-1.379	-3.039

*B *(K)	999.0	999.0	689.3	534.7	999.0	999.0	518.4	999.2

*T*_0 _(K)	145.7	145.5	169.8	185.5	146.2	145.3	189.9	148.7

*s *(mPa s(	0.29	0.43	0.32	0.36	0.18	0.33	0.16	0.70

**Table 5:** Coefficients *A*, *B*, *T*_0_, and standard deviation, *s*, from Vogel-Fulcher-Tammann equation for S2 Al_2_O_3_/EG nanofluids at different volume concentration, ***ϕ***

	*ϕ*					
	
	0.000	0.005	0.010	0.015	0.021	0.031
*A*	-3.694	-3.617	-1.558	-2.161	-2.540	-2.767

*B *(K)	999.0	999.1	493.2	616.2	745.9	847.7

*T*_0 _(K)	145.7	146.7	191.6	182.9	171.1	163.6

*s *(mPa s)	0.29	0.36	0.10	0.41	0.34	0.48

**Figure 6:**
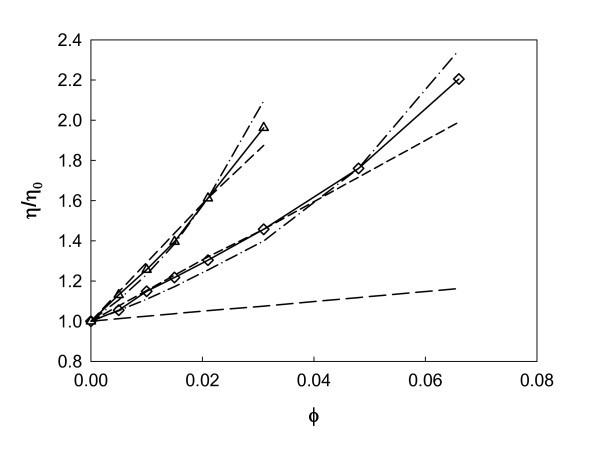
**Enhancement of viscosity increase for alumina nanofluids as a function of volume fraction of nanoparticles**. S1 (*diamond*) and S2 (*triangle*) samples. Prediction of Einstein equation (*broken solid line*), Equation 3 with *N *= 1 (*dashed line*), Equation 5, considering variable *a*_a_/*a *ratio (*dashed-dot line*), and Equation 5 considering constant *a*_a_*/a *ratio (*solid line*).

Viscosity increases with volume fraction, as expected, and this enhancement, defined as (*η*_nf _- *η*_0_)/*η*_0_, *η*_0 _being the viscosity of the base fluid, can be considered temperature-independent by analyzing Table 3. This approximation was also considered by Chen et al. [45] and Prasher et al. [47]. Thus, average viscosity increase values for each studied nanofluid were assumed over the temperature range because it allows a convenient representation of results (Figure 6). S1 and S2 samples, although sharing the same nature and nanoparticle concentration, exhibit remarkably different viscosity enhancements, and the difference between both trends is increased with concentration, as can be observed in Table 3 or in Figure 6. S2 samples, whose average nanoparticle size is smaller, show a significantly larger viscosity than S1 samples. These variations must be carefully considered because they indicate that the differences in size or aggregation of the nanoparticles used to produce a nanofluid have a determining influence on its viscosity. This effect should be analyzed when any practical application of the nanofluid is envisaged. As an example, at 10% weight fraction, viscosity enhancements of 46% and 96% are obtained for S1 and S2 samples, respectively, while for S1 samples, enhancements from 5% up to more than twice the base fluid value for the lower and higher volume fractions are found. The influence of particle size in a colloid viscosity is well known [48] due to effects, as for instance, of the electric double-layer repulsion.

This viscosity enhancement of nanofluids with volume fraction has already been cited in literature as noted above, but again, there is no agreement about the underlying physical reasons for this behavior. Several authors have proposed semi-empirical equations to describe the enhancement of the viscosity of concentrated suspensions (*η*_r _= *η*_nf_*/η*_0_, where *η*_nf _and *η*_0 _are the nanofluid and base fluid viscosity, respectively) as a function of the volume fraction only, inspired by the original expression of Einstein [49] who derived a linear relation. This classical approach largely underestimates the usual nanofluid viscosities. Nevertheless, many authors followed this approach, proposing similar correlations with variable degree volume fraction polynomials, as in the case of Chow [50]. This author presented a theory to describe the viscosity of concentrated dispersions of arbitrary-shaped particles. For the simplest case of spherical monodisperse particles, the author demonstrates that a good approximation of the enhancement of the viscosity can be obtained with a polynomial expansion of volume fraction, as follows:(3)

where *N *is the degree of the expansion and *C_i _*are coefficients. Equation 3 reduces to the well-known Einstein [49] expression for dilute dispersion viscosity if *N *= 1 and *C*_1 _= 2.5. As commented, Figure 6 shows that the Einstein relation underestimates the enhancement of the viscosity, especially at higher concentrations. A fit of Equation 3 to experimental viscosities was considered, with *N *= 1, yielding *C*_1_values of 15.2 and 29.2 for S1 and S2 samples, respectively. These correlations are also shown in Figure 6, and absolute average deviations of 2% were reached for both sets of samples. No significant improvements have been obtained if *N *= 2 is considered in Equation 3. As an alternative approach, we have applied the following semi-empirical relationship for viscosity of dispersions covering the full range of particle volume fraction obtained by Krieger and Dougherty [51]:(4)

where *ϕ*_m _is the maximum particle volume fraction and [*η*] is the intrinsic viscosity, whose typical value for monodisperse suspensions of hard spheres is 2.5. Then, if nanoparticles in nanofluids are assumed to form aggregates, and hydrodynamic forces are considered insufficient to break the structure of aggregates into isolated particles, the flow of such stable aggregates must be taken into account. Considering the effects of variable packing fraction within the aggregate structure, an approximate expression for the nanofluid enhancement of the viscosity can be derived [45]:(5)

where *a*_a _and *a *represent the average radius of the aggregates and single particles, respectively. This theory attributes the viscosity enhancement of a nanofluid only to the aggregation state of the nanoparticles. Assuming as Chen et al. [45] Newtonian behavior for EG-based nanofluids and the enhancement of the viscosity depending on particle concentration in a nonlinear manner but independent of temperature, we considered the size of the aggregates dependent on nanofluid concentration. Thus, a value of the ratio *a*_a_/*a *was computed in Equation 5 for each nanofluid concentration. This calculation offers ratio values from 3 to 4 for S1 samples, whereas these fitted parameters goes from 5.2 to 6.5 for S2 samples. The value of this parameter is always higher in S2 than in S1 sample, but this difference decreases when concentration rises. The goodness of this Equation is plotted in Figure 6, and deviations lower than experimental uncertainties are obtained, showing the suitability of the proposed theory to describe the viscosity for these EG-based nanofluids.

Finally, Equation 5 was applied using the size of the aggregates as independent of the nanofluid concentration. This way, when this equation is fitted to experimental viscosities of this work, ratios of *a*_a_*/a *of 3.2 and 5.5 are found for S1 and S2, respectively, yielding viscosity absolute average deviations of 3% and 2% for both fluids. According to this theory, the aggregation phenomenon is more relevant for smaller particles dispersions as it has been found to occur as the result of this calculation. The results from Equation 5 using only one parameter for all S1 and S2 samples are also plotted in Figure 6. With this model, aggregation alone might not be enough to describe as well the behavior of viscosity at higher concentrations, so in this case, other variables should be taken into account.

## Conclusions

Thermal conductivities and viscosities of Al_2_O_3 _in ethylene glycol nanofluids have been determined experimentally as a function of volume concentration and temperature. Two different types of samples were considered for viscosity, with nominal particle sizes of 43 and 8 nm, denoted here as S1 and S2, respectively, while S1 samples were considered for thermal conductivity studies. It has been found that the thermal conductivity and the viscosity increase with the concentration of nanoparticles, whereas when the temperature increases the viscosity diminishes and the thermal conductivity rises. Enhancements up to 19% and more than twice the value of the base fluid were found for thermal conductivity and viscosity, respectively. Viscosity increases as particle size decreases, following the expected classical behavior for dispersions. These large differences on viscosity depending on particle size must be taken into account for any practical application. We have used the Maxwell model to predict the thermal conductivities, finding that the Maxwell method overpredicts these experimental values. The Vogel-Tammann-Fulcher method was applied to the experimental viscosity data, finding good agreements and showing that this correlation with temperature is suitable also for nanofluids. Among the methods to describe the viscosity trend with the volume fraction of nanofluids, that from Krieger and Dougherty, which attributes the viscosity enhancement of a nanofluid only to the aggregation state of the nanoparticles, gives excellent results in this particular case, so here there is no need to consider the influence of other variables, as for instance sample polydispersity.

## Competing interests

The authors declare that they have no competing interests.

## Authors' contributions

MJPG performed the nanofluid samples characterization and experimental measurements, LL implemented the thermal conductivity experimental setup, performed data correlation, statistical analisys of data and coordinated the redaction of the manuscript, JLL contributed with the selection of the most suitable theoretical methods, MMP conceived of the study, and participated in its design and coordination. All authors read and approved the final manuscript.
